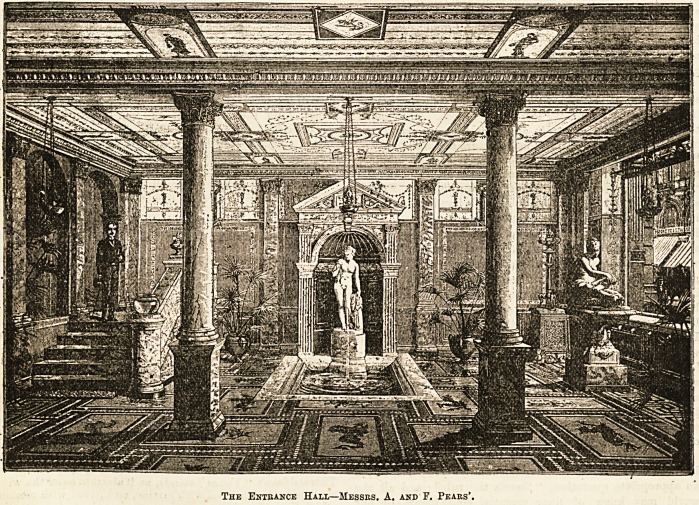# The Cult of Cleanliness

**Published:** 1889-02-16

**Authors:** 


### THE CULT OF CLEANLINESS.

A RECENT distinguished visitor to our island expressed the opinion that had he judged of British industries from the advertisements at railway stations, he would have guessed the chief of them to be the manufacture of mustard, for Colman's mustard, printed in black on a yellow ground, and Keen's mustard, yellow on black, met his eye on entering or leaving every one of them. Had Dr. Holmes continued his researches further, had he studied the interior of railway carriages, omnibuses, tramway-cars, and, above all, exhibitions, he would have been impressed with the notion that the British nation devoted all its energies to the worship of soap. Soap of all sorts—soap for the toilet, the bath, and the laundry; soap that indignantly declares, by the voice of its Monkey trade-mark, that it "won't wash clothes;" and soap that insinuates that it will perform that feat without more than the laziest aid from human hands; soap in powder, soap in balls, and soap in leaves; soap that will make everything it touches almost as good as new, or better; Pears' soap, Hudson's soap, Sunlight soap, Sapolio, and the British public knoweth how many others; soaps of every kind press their claims upon us, testifying to the national cult of cleanliness, and the greatest of these is Pears'.

Considering how peculiarly British a manufacture that of soap now is, it seems a little strange to reflect that we borrowed the art of making it from the French. That was in the twelfth century, however, and since then our neighbours have lagged behind us both in manufacture and consumption. Soap, good soap, and plenty of it, is not such an absolute necessary of life to the Frenchman as to the Englishman; the "*prêtes-moi ton savon, je te prie*," with which the French schoolboy appeals to his English comrade implies that it is a luxury, precious, indeed too precious for common use. The French had inherited the art of soap-making from their ancestors the Gauls, who had learned it from the Romans, who probably were taught it by the Greeks, who in turn had gained their knowledge from the wise old Egyptians. The history of soap goes back from the new civilisations to the old by the same route as so many other of the comforts of life, finding its cradle in the bosom of old Nile. In Egypt there existed lakes whose water were impregnated with *natron* or soda, which the inhabitants learned to employ for cleansing purposes; but it was harsh and destructive in its action, and some thoughtful chemist, some genius who has attained the immortality of oblivion—that surest proof that his ideas have become part and parcel of the common stock of knowledge—hit upon the notion of modifying the causticity of the soda by combining it with some oily substance.

This is what soap fundamentally is—a chemical combination of fat and soda. Fundamentally—yes; but, to use an appropriate quotation, "there's the rub"; and all the world may know of these essential constituents of our popular detergent without being able to make a cake of soap for the meanest domestic use. The manufacture of a small quantity of soap, according to the formula in his text-book is a favourite experiment of the amateur chemist. It is admirable soap to describe in a mystic equation, but if he is rash enough to wash his face with it, that amateur chemist is apt to repent the deed. The soap has cleansing properties certainly, but these seem as nothing in comparison with its potency in burning and peeling his skin; and while smarting under the results of his rash attempt, he develops a respect, unfelt before, for the mysteries of manufacture. He has learned that though knowledge may be power, to know the component ingredients of any substance is not identical with the power to mingle them by such methods and in such proportions as will produce a satisfactory result. It is the lesson of life—that an ounce of practice is worth a pound of theory; as true a lesson as that other—that life is a bubble, tinted rainbow-like with hope as it floats upward in the sunlight, but quickly vanishing away; and both may be learned from soap.

The first is decidedly the more useful lesson, but perhaps the second is more familiar at present to the public, which sees everywhere the reproductions, large and small, of a certain picture representing a golden-haired boy watching, with all the anxious seriousness of childhood, the progress of the bubble that has just floated off from the pipe in his hand. It is a lovely picture, one of the most attractive that even Millais has ever done; and there are those, of super-sensitive refinement, who mourn that such a work of art should help to advertise the productions of a firm of soap-makers. Why? we should like to know. Is art a thing so far removed from daily life that the artist and the manufacturer must for ever stand apart? It is not thus that nations become artistic. Nothing, believe us, oh! great but unknown masters, who pride yourselves on not being appreciated by the vulgar, tends more to keep the populace truly vulgar, than your determination that your works shall not in any way appeal to their sympathies and comprehension. The best work may be spent, and profitably spent, on the simplest subjects; and the Piccadilly shopmen, who as yet do not follow the progress of the pictures of the year on their way to Burlington House, after the fashion of the much-quoted Florentine apprentices of old, are more likely to do so when painters choose subjects which do not involve the said shopmen in the necessity of studying "Lemprière" or the "Niebelungenlied" before they can know what the pictures are about. For our own part, we think that the artist does not derogate from his calling in painting for the manufacturer, and that the manufacturer does something for art as well as for his trade when he spreads through the length and breadth of the land good chromo-lithograph copies of such a picture as "Bubbles."

The original picture may be seen by those who have the opportunity to penetrate to the interior of the central depôt of "Pears' Soap" in New Oxford Street. It is worth seeing, that establishment, which occupies the whole height and depth of a large building. From the busy street, with its hansoms and omnibuses, and other evidences of the modern spirit, the visitor goes straight into a hall that resembles nothing more modern than the time of the Cæsars. As a matter of fact, it is an accurate reproduction of a Pompeian court. The floor is of mosaic, inlaid with patterns of classic vases, intermixed with dolphins and dragons. The walls are painted in those clear bright colours that somehow manage to avoid being harsh or crude, and the pillars and lintels of the doorways are of carved marble of various tints. From the painted ceiling hang lamps of classic shape, from which the sole anachronism in the apartment, the electric light, sheds its radiance on the pure white of various statues. Mr. Lawes' "Bather" is stretched before the window, and a copy of Thorwaldsen's "Venus" stands, as if about to enter the wide shallow bath, bordered with marble, and paved with mosaic, which lies at her feet, or perhaps she is only looking at the slender fountain that rises in the middle of it, or the gold fish that swim in its basin.

From the hall a marble staircase leads to the strictly business part of the premises above. Everything is modern enough here—the polished desks, the busy clerks, the sound of the constantly-employed type-writer. Passing through this one goes into the waiting-room, a very comfortable apartment, where on two large easels are displayed "Bubbles," and the companion picture, "Les Bulles de Savon," commonly called "More Bubbles," by Edouard Frère. Besides these there are hung on the walls other works of art—a pretty little girl reading a pretty large book, and two studies by Trood, one representing a monkey shaving a dog, and the other a monkey, more mischievous still, intent on washing a cat. All these pictures have been utilised for the purposes of advertisement, as have others "too numerous to mention," to use the common phrase. Most people know that picture by Stacy Marks, of the two monks at their toilet which bears the legend, "Cleanliness is next to Godliness," the far-famed and ubiquitous "Dirty Boy," the babies of all ages and countries that are expressing their appreciation of Messrs. Pears and their works, including that self-willed infant whose expression emphatically confirms the appended declaration that, "he won't be happy till he gets it."

You may see copies of all these in a large room that resembles a well-stocked wholesale printers' warehouse. These are the small advertisement cards; larger ones for shop windows and hoardings dwell in other apartments, and one room is entirely devoted to the wooden cases in which they are sent out. Truly everything is done on a colossal scale here. Going up to a higher storey we come on a dark chamber where, through two lanterns that were once the property of the Polytechnic, the oxy-hydrogen light displays some of the most popular advertisements to an admiring crowd below. On going down to the lower floor we come to the electric chamber, where is generated the light that illuminates the establishment and the force that works the lift. Here, too, is the strong room—a Blue Beard's chamber, with a grated door, dark till that door is opened, when it immediately becomes luminous with electric light. There are no horrors which is that ballad which tells of the wondrous transformation it wrought on "Bishop Q., of Wangaloo, in unpacific seas."

How is it then that this soap, of which the things we have described are but the adjuncts and accessories, has attained its wide and lasting notoriety? There are scores of toilet soaps in the market, dozens of transparent soaps, yet none so popular as this. Some people, indeed, who have been unlucky in their experiments, declare that they hate transparent soaps, that they are sticky and nasty, and don't cleanse at all. Bad transparent soaps are certainly very bad indeed. They are made with cocoa-nut oil, which, in opposition to the popular belief that "oil and water won't mix," is capable of taking up about 80 per cent. of water. The transparency is attained by the addition of glycerine, sometimes as much as 25 per cent., and all the cleansing power is left to the work of a very harsh amount of caustic soda. Nobody wants to buy 40 or 50 per cent. of water in their soap, and those who unwittingly do so are apt to look upon all transparent soaps with disfavour.

The Entrance Hall—Messrs. A. and F. Pears'.

here, however, only the books of the firm and two moderate-sized cases, which contain something like two thousand pounds' worth of otto of roses. On this floor, too, some soap is kept in stock—not much, though it seems a good deal to an outsider, but just enough to supply the day's needs of London alone. The rest of the world—India, America, Australia, and the rest—get their goods direct from the works at Isleworth, which cover two acres of ground, and give employment to 500 people.

The firm has its archives too—the advertisements in the *Times* of the Waterloo year, another in *Bell's Messenger* of a slightly later date, where it stands near the announcement of the merits of "Dr. James's Analeptic Pills." The "analeptic pills," whatever they were, have gone the way of many patent medicines, but Pears' soap survives. The muse, too, has been pressed, or rather, has willingly gone into its service. It was of his own free will that Sir Theodore Martin, in the "Bon Gaultier Ballads" described Venus as rejoicing in the possession of "cakes of Pears' transparent soap;" and many songs have been sung in its praise, not the least interesting of

Good transparent soap, such as Messrs. Pears', is made differently. The foundation of it is good ordinary soap, cut into shavings, which, by giving the carbonic acid in the air free access to it, neutralises the causticity of the soda in it. These shavings are then treated with alcohol, which helps still further to get rid of all superfluous soda. When the alcohol evaporates, transparent soap is left—transparent by reason of the absence of all unnecessary or deleterious ingredients.

The testimony borne to the excellence of Pears' soap by such men as the late Sir Erasmus Wilson—whose commendation was entirely spontaneous, for he had no interest in the firm, and did not even know its principals; by Professor John Attfield, of the Pharmaceutical Society; by Sir Charles Cameron, Professor of Chemistry and Hygiene in Dublin; and Stevenson Macadam, Lecturer on Chemistry in the Surgeons' Hall, Edinburgh, all speaking of its excellence and purity—give the scientific explanation of that enormous popularity which has elevated Messrs. Pears to the position of high priests of the cult of cleanliness.

## Figures and Tables

**Figure f1:**